# A Statistical Shape Model of the Morphological Variation of the Infrarenal Abdominal Aortic Aneurysm Neck

**DOI:** 10.3390/jcm11061687

**Published:** 2022-03-18

**Authors:** Willemina A. van Veldhuizen, Richte C. L. Schuurmann, Frank F. A. IJpma, Rogier H. J. Kropman, George A. Antoniou, Jelmer M. Wolterink, Jean-Paul P. M. de Vries

**Affiliations:** 1 Department of Surgery, Division of Vascular Surgery, University Medical Center Groningen, 9713 GZ Groningen, The Netherlands; r.c.l.schuurmann@umcg.nl (R.C.L.S.); j.p.p.m.de.vries@umcg.nl (J.-P.P.M.d.V.); 2 Multimodality Medical Imaging Group, Technical Medical Centre, University of Twente, 7500 AE Enschede, The Netherlands; 3 Department of Surgery, Division of Trauma Surgery, University Medical Center Groningen, 9713 GZ Groningen, The Netherlands; f.f.a.ijpma@umcg.nl; 4 Department of Vascular Surgery, St. Antonius Hospital, 3435 CM Nieuwegein, The Netherlands; r.kropman@antoniusziekenhuis.nl; 5 Department of Vascular Surgery and Endovascular Surgery, The Royal Oldham Hospital, Manchester University NHS Foundation Trust, Manchester M13 9WL, UK; antoniou.ga@hotmail.com; 6 Division of Cardiovascular Sciences, School of Medical Sciences, The University of Manchester, Manchester M13 9PL, UK; 7 Department of Applied Mathematics, Technical Medical Centre, University of Twente, 7500 AE Enschede, The Netherlands; j.m.wolterink@utwente.nl

**Keywords:** analysis, principal component, statistical shape model, aortic aneurysm, abdominal, endovascular aneurysm repair, aneurysm neck morphology

## Abstract

Hostile aortic neck characteristics, such as short length and large diameter, have been associated with type Ia endoleaks and reintervention after endovascular aneurysm repair (EVAR). However, such characteristics partially describe the complex aortic neck morphology. A more comprehensive quantitative description of 3D neck shape might lead to new insights into the relationship between aortic neck morphology and EVAR outcomes in individual patients. This study identifies the 3D morphological shape components that describe the infrarenal aortic neck through a statistical shape model (SSM). Pre-EVAR CT scans of 97 patients were used to develop the SSM. Parameterization of the morphology was based on the center lumen line reconstruction, a triangular surface mesh of the aortic lumen, 3D coordinates of the renal arteries, and the distal end of the aortic neck. A principal component analysis of the parametrization of the aortic neck coordinates was used as input for the SSM. The SSM consisted of 96 principal components (PCs) that each described a unique shape feature. The first five PCs represented 95% of the total morphological variation in the dataset. The SSM is an objective model that provides a quantitative description of the neck morphology of an individual patient.

## 1. Introduction

Endovascular aortic aneurysm repair (EVAR) is the preferred treatment for the exclusion of an abdominal aortic aneurysm (AAA) in the presence of suitable aorto-bi-iliac anatomy [1,2]. EVAR is associated with lower 30-day mortality and morbidity rates and shorter intensive care and hospital stay compared to open surgical procedures [3,4]. Hostile aortic neck characteristics, such as short length (<1 cm), severe supra- (>45°) and infrarenal (>60°) angulation, conicity, and diameter > 30 mm, have been associated with higher rates of type Ia endoleak, reintervention, and aneurysm-related mortality [3,5,6,7,8,9,10,11]. For more than two decades, these anatomical characteristics have been treated as separate measurements, resulting in an oversimplified description of the complex 3D morphology of the aortic aneurysm neck [12].

Currently, there is no consensus on the definition of hostile aortic neck morphology and how the individual neck characteristics or a combination of characteristics influence post-EVAR complications [13]. A more objective and personalized perspective on the 3D neck shape could provide more information that vascular surgeons can use during the decision making for conventional EVAR, fenestrated or branched-EVAR, open surgical repair, or a watchful waiting policy.

We hypothesize that an objective and patient-specific determination may be provided by investigating the 3D morphological infrarenal aortic neck shapes in a given dataset. This can be accomplished by means of a statistical shape model (SSM). An SSM is a mathematical technique that models the shape variation of an anatomy of interest in a population. For this, a principal component analysis (PCA) is performed, which returns linearly independent components describing the variation of the shape in the population, capturing potential interactions between morphological characteristics in these different components [14]. SSMs are widely applied for medical purposes, such as computer-assisted surgical planning tasks [15,16,17].

The SSM distinguishes unique shape components into a morphological model in such a way that the aortic neck morphology of a single patient can be reconstructed from a combination of these separate components. The shape components obtained from the SSM are not necessarily the same as the present known neck characteristics since a shape component might be a combination of these characteristics, such as angulation and neck diameter.

This study aims to identify the 3D morphological shape components that describe the infrarenal aortic neck of a population of AAA patients who were treated with an EVAR procedure without proximal neck-related complications at the first follow-up scan.

## 2. Materials and Methods

### 2.1. Study Population

A retrospective observational imaging study was conducted to determine the shape variation of the infrarenal aortic neck morphology using an SSM. In total, 98 patients from an existing consecutive dataset who underwent a primary elective EVAR procedure with the Endurant II or IIs device (Medtronic Inc., Santa Rosa, CA, USA) between January 2014 and December 2017 were evaluated for inclusion (registration number 00287, 2021). The study was approved by the institutional review board of both hospitals according to the Declaration of Helsinki. Informed consent was waived according to institutional policy on retrospective research.

CT scans were acquired at the St. Antonius hospital, Nieuwegein, The Netherlands (57 patients), and the Royal Oldham Hospital, Manchester, United Kingdom (41 patients). Inclusion criteria for the existing dataset were a technically successful EVAR procedure to treat an infrarenal AAA with the Endurant II or Endurant IIs endoprosthesis and the availability of a CTA scan within 12 months pre-EVAR and a CTA scan within six months post-EVAR. Exclusion criteria were treatment of a symptomatic or ruptured AAA; adjunct proximal fixation, such as cuffs or endoanchors; CT scans with insufficient contrast for assessment in a vascular workstation; and intentional low (>5 mm) positioning of the endoprosthesis relative to the lowest renal artery. An additional exclusion criterion for this study was the presence of a type Ia endoleak on the completion angiography or on the first follow-up scan.

### 2.2. Measurements in Vascular Workstation

In each preoperative CT scan, the center lumen line (CLL) and a triangular surface mesh of the aortic lumen were semi-automatically obtained by an experienced researcher using a 3mensio Vascular Workstation (version 9.1 SP2, Pie Medical Imaging BV, Maastricht, The Netherlands). 3D coordinates were placed at the orifice of the lowest renal artery (LRA) and at the end of the aortic neck, which was defined as a 10% increase in the diameter compared to the lowest renal artery baseline. The center lumen line, the mesh of the aortic lumen, and the 3D coordinates were exported to MATLAB 2018a (The MathWorks, Natick, MA, USA) for further analysis. Dedicated software was designed in MATLAB with a pipeline for preprocessing of the data, alignment of the infrarenal neck meshes, and finally, PCA and SSM.

### 2.3. Preprocessing and Aortic Neck Alignment

**Figure 1:**
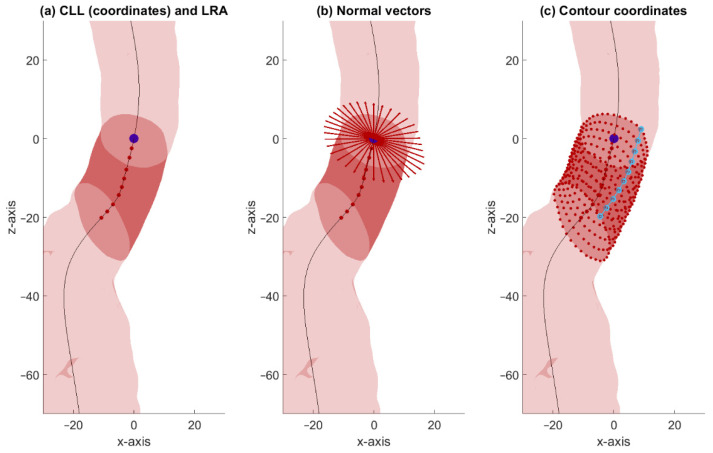
Visualization of the parameterization of one patient. The surface of the aortic lumen is visible in light red, and the surface of the aortic neck is visible in dark red. (**a**) The CLL (black line), ten equidistantly spaced contour points on the CLL (red dots), and the point on the CLL that corresponds to the projection of the origin of the lowest renal artery coordinate (blue dot); (**b**) for the first CLL coordinate, 36 normal vectors are displayed; (**c**) the 360 intersections of the normal vectors with the mesh are displayed as red dots. Ten coordinates (light blue dots) at the same orientation were longitudinally rearranged at equal distance (light blue line).

The CLL between the lowest renal artery baseline and the distal end of the aortic neck was divided into ten equidistant segments. For each segment, 36 normal vectors were calculated with 10° intervals. The intersection of the vectors with the surface mesh of the aortic lumen was calculated using the method developed by Tuszynski [18]. This resulted in 36 contour points in a ring around the centerline over the contour of the aortic neck for each segment and a total of 360 contour points for every patient (Figure 1).

After parametrization, an additional preprocessing step was implemented. To prevent the crossing of adjacent contours, we rearranged the 10 parallel contour points from each contour line at equal distances along the longitudinal axis through linear interpolation. In Figure 1c, an example of such a longitudinal line including the 10 parallel contour points is shown. Subsequently, the 360 contour points were translated so that all infrarenal aortic necks were aligned based on the lowest renal artery baseline.

These steps resulted in smooth contour lines at equal distances over the length of the infrarenal aortic neck that are suitable for point-to-point correspondence. The contour points obtained after the alignment step were used as input for the PCA.

### 2.4. Principal Component Analysis and Statistical Shape Modelling

The SSM was adjusted from the work of Manu, who had previously created an open-source SSM in MATLAB for clinical purposes, and applied to the scapula, the knee, and the femoral head-neck junction [19,20,21,22]. The SSM calculates the 3D mean shape of the infrarenal aortic neck by averaging the paired contour points of all patients in the dataset. The contour points of the individual patients are compared to this mean shape by singular value decomposition, resulting in a set of eigenvectors and corresponding eigenvalues for each neck morphology. These eigenvectors and eigenvalues are used to compute the principal components (PCs) that describe the unique components of the shape variation and their variance. In Appendix A, a description of the mathematical background of PCA is provided.

**Figure 2:**
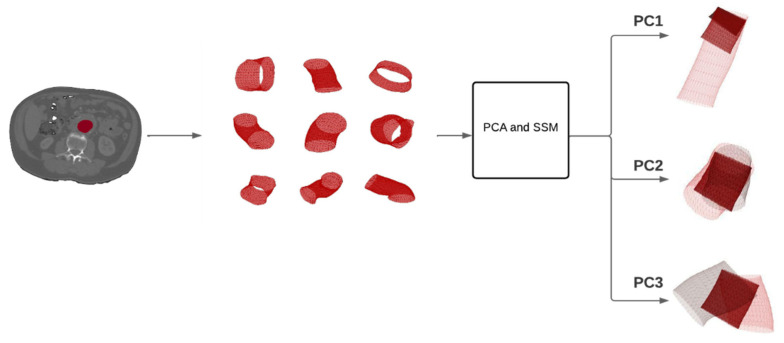
Workflow of the process from a CT scan to the principal component analysis (PCA) and statistical shape model (SSM) results. The first step is to segment the aortic neck lumen in the CT scan (red segmentation). Nine random samples, taken from the dataset consisting of 97 patients, are displayed in the second box to show the anatomical variation. The third box represents the mathematical procedures applied to perform a PCA and obtain the SSM. On the right, three visualizations, corresponding to the first three PCs, with the results of the SSM, are shown.

The shape variance of each principal component (PC) was visualized as a mesh of the mean shape ± 3  standard deviations (SD) for normally distributed PCs, describing 99.7% of the shape variation of the studied population. Figure 2 shows the workflow from the segmentation of the aortic neck lumen on the CT scan to the output of the PCA and SSM in terms of PCs 1–3.

### 2.5. Evaluation

The quality of the SSM was quantitatively evaluated by calculating the compactness, generalization, and specificity [23,24]. The compactness curve describes how many PCs are needed to describe a certain amount of variation in the dataset. The generalization ability describes how well the SSM reconstructs a new and unseen shape, i.e., the anatomy of a random patient [23]. The generalization ability was conducted by a leave-one-out cross-validation method. This means that we excluded one patient from the dataset and built a statistical shape model using the 96 remaining aortic neck shapes. We then used this SSM to reconstruct the shape of the left-out patient and quantified the difference between the original shape and the reconstruction with the root mean square error (RMSE). This process was repeated for all patients.

Specificity was assessed to evaluate the model’s performance to create valid random reconstructions of an aortic neck that matches shapes in the original dataset [23]. The error between the artificial shape and its most similar shape from the original dataset was also computed by means of RMSE. A detailed, mathematical description of the evaluation steps is provided in Appendix B.

## 3. Results

**Figure 3:**
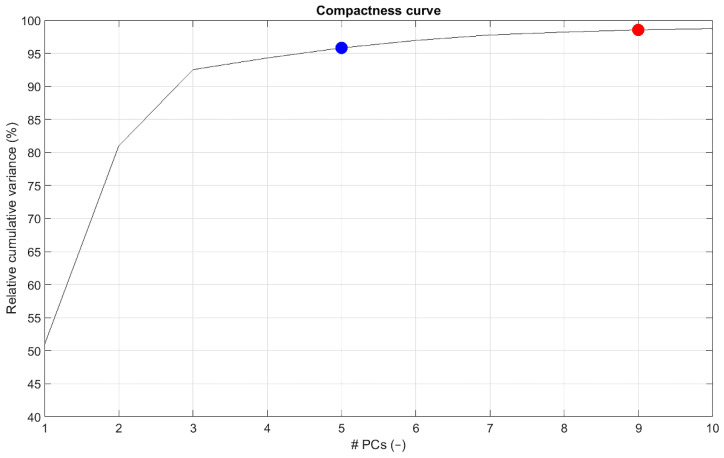
Compactness curve of the statistical shape model (SSM) that shows the cumulative morphological variation that is described by the principal components (PCs). The blue dot indicates 95% of the total variation by the first five PCs; the red dot indicates 98% of the total variation described by the first nine PCs.

In total, 97 patients were included in this study, of which 84 patients were male (87%), and the mean age was 75 ± 7 years. Ninety-three of ninety-seven (96%) patients were treated inside Instructions For Use (IFU) of the Endurant endograft. As default for any SSM, 96 (the number of patients-1) PCs were identified. The first five and the first nine PCs described 95% and 98% of the total morphological variation in the dataset, respectively. The first PC described 51% of the total variation, PCs 2–5 described 30%, 12%, 2%, and 2%, respectively (Figure 3). The research team (W.V., R.S., F.I.J., J.W., and J.V.) evaluated the first nine PCs visually to check if these components described relevant and realistic anatomical variations. Based on the subjective interpretation and the fact that these shape components described 95% of the total variation, we determined that the first five PCs were the main components describing the shape variation.

**Figure 4:**
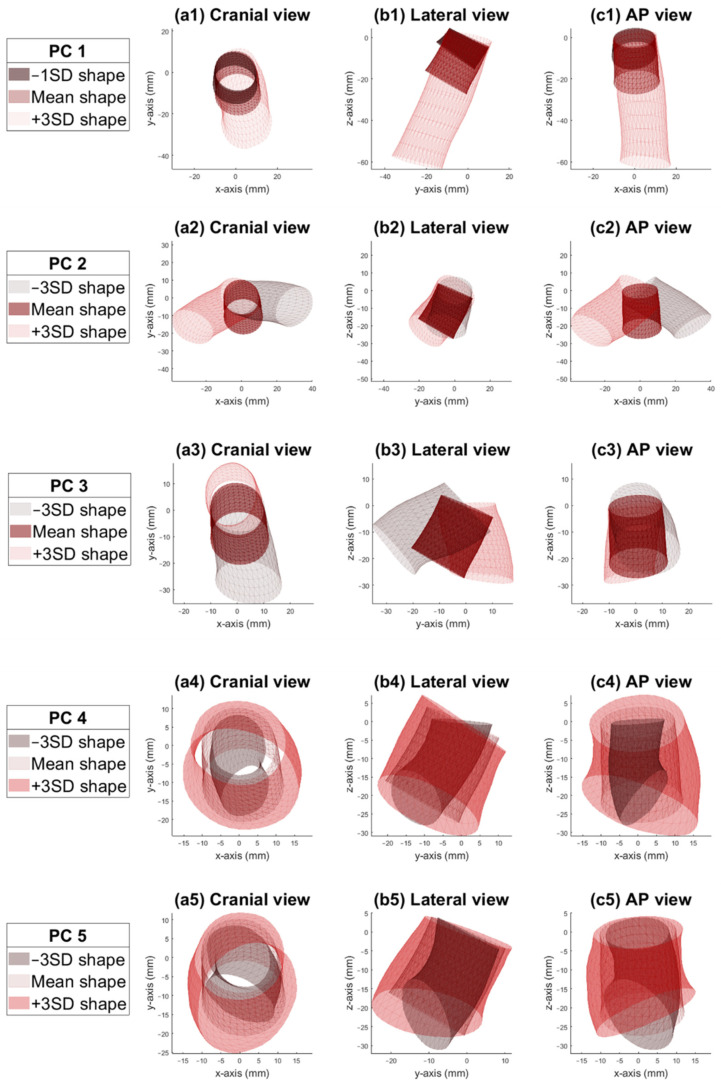
Visualization of shape variation of the abdominal aortic neck for the first five principal components (PCs). PC 1 describes 51% of the total shape variation, which mainly is variation in neck length, with −1SD representing the shortest neck in the dataset and +3SD the longest neck. PC 2 describes 30% of the total variation, which is mainly deflection to the left and right. Deflection in the right direction is oriented more anteriorly compared to deflection to the left. PC 3 describes 12% of the total variation, mainly deflection in the anterior direction. PC 4 describes 2% of the total variation but is an important component, as it describes mainly the neck diameter. PC 5 also describes 2% of the total variation, which is mainly deflection of the distal neck end.

Figure 4 shows the first five PCs of the infrarenal aortic neck shape variation. None of the formally known hostile neck characteristics were used as input for the SSM; only the infrarenal aortic neck shapes in the dataset were used as input for the model.

The first PC describes mainly a variation in neck length (Figure 4, first row). The first PC was not normally distributed, as the neck length was also not normally distributed in the dataset. Therefore, the visualization of the shortest neck was set to −1SD instead of –3SD, which corresponded with the distribution of the dataset. In Appendix C, this is explained in further detail. The second PC mainly describes deflection to the left and right (Figure 4, second row), the third PC mainly deflection in the anterior direction (Figure 4, third row), the fourth PC mainly describes neck diameter (Figure 4, fourth row), and the fifth PC mainly the deflection of the distal neck end (Figure 4, fifth row). PCs 6 to 9 describe minor shape variations that may not have a clear clinical significance but are needed to complement the aortic neck shape to have a generalizable and robust model.

The accuracy of the SSM to reconstruct a given new patient-specific shape, the mean generalization, of the SSM with the first five PCs is 3.6 (95% CI 3.2–3.9) mm. The mean accuracy with nine PCs is 3.3 (95% CI 2.9–3.8) mm. The lowest error value was found with an SSM that includes nine PCs.

**Figure 5:**
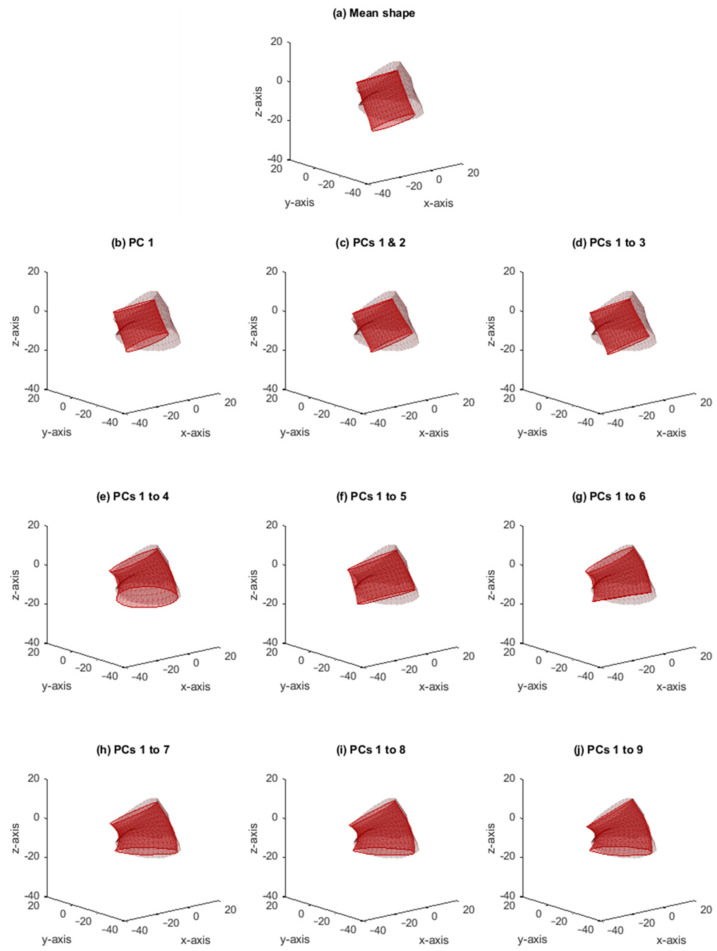
Example of a patient’s original shape (brown mesh) and the reconstructed shape (red mesh), starting with the mean shape as the reconstructed shape (**a**). In each of the subplots (**b**–**j**), the number of included principal components (PCs) in the statistical shape model (SSM) is increased by one. The root mean square error (RMSE) of principal component (PC) 1 is 6.5 mm, whereas the mean RMSE of PCs 1 to 9 is reduced to 2.5 mm.

Figure 5 shows an example of an individual patient anatomy and its reconstructed shape by the SSM. This figure shows what the contribution of each PC is to reconstruct a given shape and how the combination of these PCs in the SSM is able to recreate a patient-specific aortic neck. With each addition of a PC representing a morphological shape component, the model is more precise in reconstructing a patient-specific shape. PC 4, for example, adjusted the neck diameter and added deflection, which is visible in the shape change in Figure 5d,e. The generalization error for this specific patient started with 6.5 mm for one PC and was 2.5 mm for nine PCs.

The specificity of the SSM, the ability of the model to create an artificial and realistic shape, was 4.8 (95% CI 4.7–4.8) mm, including five PCs and 5.2 (95% CI 5.1–5.2) mm, including nine PCs.

## 4. Discussion

In this study, we developed an SSM of the infrarenal aortic neck shapes to visualize and quantify the morphological shape variation of the infrarenal aortic neck of patients treated with standard infrarenal EVAR and to obtain a quantitative description of the neck morphology of an individual patient.

Current measurements of neck characteristics, such as neck length, diameter, and supra- and infrarenal angulation, are a simplification of the true morphological shape. It is still unknown if a combination or which combination of neck characteristics most influences successful EVAR outcomes [13]. The proposed SSM with five morphological shape components includes the combination of variation in length, deflection, and diameter and provides insight in anatomical features that are not captured by the conventional measurements. In addition to simplified centerline measurements, the SSM is able to reconstruct a complete infrarenal neck shape from patient-specific morphological shape components.

The current SSM uses a digital twin of the aorta as input. The SSM describes the variation of anatomical features that are present in the population of AAA patients. Parallel to this concept, digital models built with the help of artificial intelligence from preoperative aortic CT scans enable preoperative sizing in complex endovascular repair [25]. The development of automated segmentation and supervised deep learning may enrich the current SSM models with more detailed aortic segmentation [26]. Both techniques should be embraced by modern endovascular specialists taking care of (complex) AAA patients.

In the vascular field, Liang et al. implemented an SSM to associate morphological shape features of the ascending thoracic aortic aneurysms with the risk of rupture [27]. De Bruijne et al. created an SSM of the abdominal aortic aneurysm as a prerequisite for automated segmentation [28]. An SSM of the infrarenal neck of the abdominal aortic aneurysm, which is most important for sealing of the endograft, has not been developed yet.

A significant challenge of building an SSM is in obtaining point-wise alignment between input shapes. In lieu of a large set of well-defined anatomical landmarks on the aortic aneurysm neck, here, we used a tubular parametrization [29]. For the alignment of shapes in space, we chose the LRA as the anchor point. Therefore, the shape variations are relative to the lowest renal artery baseline, which is most relevant for the EVAR procedure.

Each addition of a PC, to a maximum of nine PCs, results in an SSM that is better able to reconstruct the patient’s anatomy. The SSM, including nine PCs, is a robust model that includes 98% of the total shape variation in our dataset and that is able to describe new and unseen shapes.

Limitations of the SSM are that it relies on the input data and therefore on the patients in the dataset and the measurement of the lowest renal artery baseline and the distal end of the aortic neck. The dataset only included EVAR patients who were treated successfully with an infrarenal Endurant II/IIs device, with 96% of the patients treated within proximal neck-related IFU and who did not have a type Ia endoleak on the 30-days CT scan. The shape variation in the current SSM is specific for this population. Another limitation is the number of patients. Addition of more patients will make the SSM more generalizable.

In future studies, a patient-specific prediction of target apposition within the infrarenal or suprarenal aortic neck with either standard infrarenal EVAR or complex repair is desired. A next step in this direction would be to add patients with complications, such as type Ia endoleak, to the SSM and to perform a comparative study with a control cohort of uncomplicated EVAR patients. Moreover, to improve the model, it would be worthwhile to add aortic necks in the SSM that were judged not suitable for standard EVAR, such as patients with a juxtarenal AAA or patients who underwent open aortic repair due to too challenging an infrarenal neck morphology. This way, specific shape components that contribute to unanticipated loss of seal or neck anatomies not suitable for standard EVAR can be identified. As a future perspective, implementation of such a semi-automatic SSM in clinical practice, providing insight in the 3D morphology, may support endovascular aortic specialists in treatment planning.

## 5. Conclusions

In this study, an SSM of the morphological shape variation of the infrarenal aortic neck was developed of patients who were treated with EVAR for an abdominal aortic aneurysm. The SSM is an objective model that provides a quantitative description of the neck morphology of an individual patient. Nine PCs provide a generalizable and robust model to determine the morphology of the infrarenal aortic neck.

## Figures and Tables

**Figure A1:**
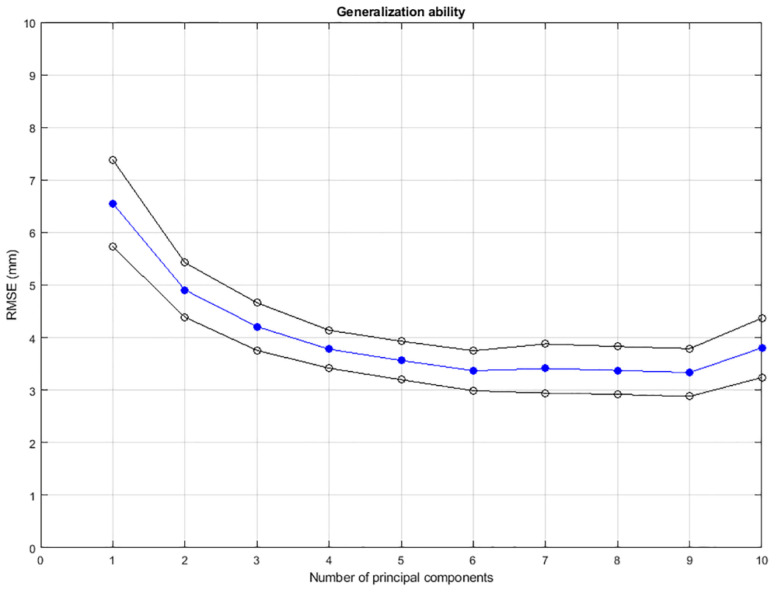
Graph of the generalization ability of the statistical shape model (SSM) by means of the leave-one-out method. The blue dots represent the root mean square error (RMSE, mm) of the reconstructed and actual shapes of *n* − 1 patients for a given number of principal components (PCs). The open, black dots represent the boundaries of the 95% confidence interval. The mean generalization of the SSM with five PCs is 3.6 (95% CI: 3.2–3.9) mm.

**Figure A2:**
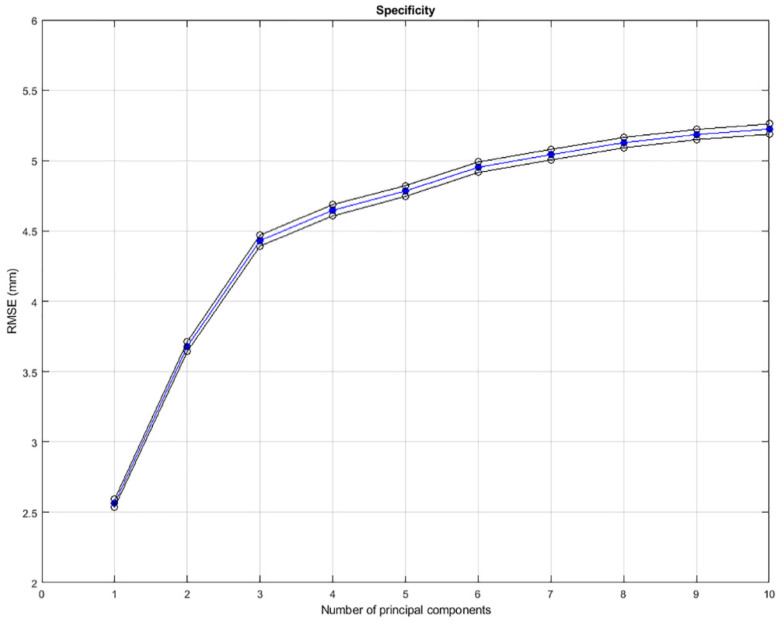
Graph of the specificity of the statistical shape model (SSM); mean specificity and the 95% confidence intervals are given. The mean specificity for five principal components (PCs) is 4.8 (95% CI: 4.7–4.8) mm.

**Figure A3:**
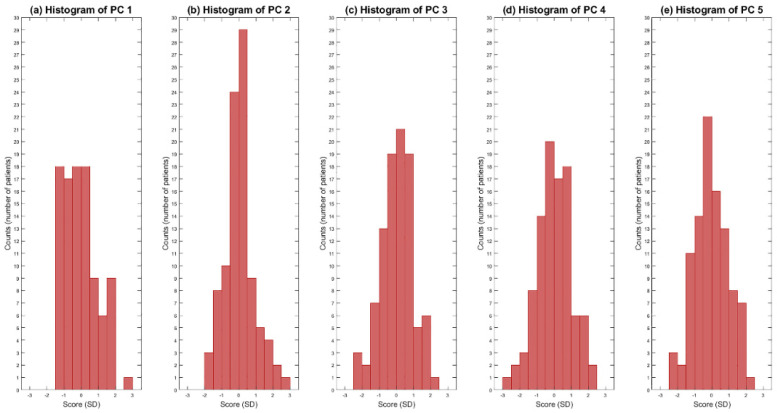
Histograms of the first five principal components (PCs). (**a**) The first principal component (PC) is not normally distributed; values < −1.5 standard deviation (SD) are not present in the given dataset. Values < −1.5 SD represent a negative neck length, which is anatomically not feasible. Therefore, only −1 SD to +3 SD deviations are shown for PC 1. (**b**–**e**) The distributions of PCs 2–5.

## Data Availability

No new data were created or analyzed in this study. Data sharing is not applicable to this article.

## Appendix A

The statistical shape model (SSM) requires a mean shape and a model of the variation in this shape. This model is based on the output of the principal component analysis (PCA). Both the mean shape and the variations are computed based on a matrix A that contains all 360 contour coordinates of all 97 patients:

(A1) $$A=\left[\begin{array}{c} \begin{array}{c} \begin{array}{ccc} x_{1}^{1} & \cdots & x_{1}^{97} \end{array}\\ \begin{array}{ccc} y_{1}^{1} & \cdots & y_{1}^{97} \end{array}\\ \begin{array}{ccc} z_{1}^{1} & \cdots & z_{1}^{97} \end{array}\\ \begin{array}{ccc} \vdots & \vdots & \vdots \end{array} \end{array}\\ \begin{array}{ccc} x_{360}^{1} & \cdots & x_{360}^{97} \end{array}\\ \begin{array}{ccc} y_{360}^{1} & \cdots & y_{360}^{97} \end{array}\\ \begin{array}{ccc} z_{360}^{1} & \cdots & z_{360}^{97} \end{array} \end{array}\right]$$

In this matrix, rows represent aligned contour point coordinates, and columns represent patients in the data set. Hence, $x_{1}^{1}$ corresponds to the first x-coordinate of the first patient, and $z_{360}^{97}$ corresponds to the last z-coordinate of the last patient. We first computed the mean shape from matrix A as $\;\bar{a}=\frac{1}{N}\overset{N}{\underset{i}{\sum }}a_{i}$, where ai corresponds to a column in A, i.e., the vector of 1080 (360 × 3) contour points for one patient.

The mean shape was used to zero-center all samples in order to compute residuals:

(A2) $$residuals=\frac{\left[\begin{array}{c} \begin{array}{c} \begin{array}{ccc} x_{1}^{1} & \cdots & x_{1}^{97} \end{array}\\ \begin{array}{ccc} y_{1}^{1} & \cdots & y_{1}^{97} \end{array}\\ \begin{array}{ccc} z_{1}^{1} & \cdots & z_{1}^{97} \end{array}\\ \begin{array}{ccc} \vdots & \vdots & \vdots \end{array} \end{array}\\ \begin{array}{ccc} x_{360}^{1} & \cdots & x_{360}^{97} \end{array}\\ \begin{array}{ccc} y_{360}^{1} & \cdots & y_{360}^{97} \end{array}\\ \begin{array}{ccc} z_{360}^{1} & \cdots & z_{360}^{97} \end{array} \end{array}]-[\begin{array}{c} {\bar{x}}_{1}\\ \vdots \\ {\bar{x}}_{360}\\ {\bar{y}}_{1}\\ \vdots \\ {\bar{y}}_{360}\\ {\bar{z}}_{1}\\ \vdots \\ {\bar{z}}_{360} \end{array}\right]}{\sqrt{97-1}}$$

Accordingly, singular value decomposition was performed on the residuals, resulting in two matrices: U and S. Matrix U represents the (360 × 3)–by–97 orthonormal columns, and matrix S represents the 97–by–97 diagonal. The eigenvalues are computed by squaring the values on the diagonal of the matrix S.

The eigenvectors are computed by multiplying each value of matrix U by its corresponding sign (either 1 or minus 1). Subsequently, a matrix ssmV with vectors representing the deviation from the mean shape was computed as follows:

(A3) $$ssmV=\sqrt{Eigenvalues}*Eigenvectors$$

## Appendix B

The quality of the statistical shape model (SSM) was evaluated using the compactness, generalization ability, and specificity [23]. The compactness curve describes how many components are needed to describe a given amount of variation in the dataset. The generalization ability describes how well the model does in reconstructing a given shape. It assesses the ability to describe unseen instances from outside the given dataset [23]. In order to assess the reconstructed shape, the root mean square error (RMSE) was computed between this reconstructed shape and the original shape by means of the leave-one-out method. Specificity was assessed to evaluate the model’s performance to create only valid instances, matching the shapes in the dataset [23].

The compactness was calculated as follows:

(A4) $$C\left(M\right)=\overset{M}{\underset{m=1}{\sum }}\lambda_{m}$$

with *M* representing the number of principal components (PCs) and $\lambda_{m}$ the *m*th eigenvalue [24].

The generalization ability was assessed by means of the leave-one-out method. A shape instance was kept from the dataset, and a principal component analysis (PCA) was performed on the remaining aortic neck lumen meshes in the dataset (97–1). The left-out shape was reconstructed by the SSM, with a varying number of PCs included (M). The reconstruction was calculated as:

(A5) $$x_{i}^{\prime }\left(M\right)=\bar{x}+\overset{M}{\underset{m=1}{\sum }}b_{m}\phi_{m}\;$$

with $\bar{x}$ representing the mean shape, and $b_{m}\phi_{m}$ representing the linear combination of the first *M* components of variation for the left-out shape. Parameter *b* was taken from the range $b_{m}\;\in \left[-n\sqrt{\lambda_{m}},\;n\sqrt{\lambda_{m}}\right]$, with *n* representing the values of standard deviations and $\lambda_{m}$ representing the *m*th eigenvalue [24].

The deviation between the reconstructed and actual shape was calculated by the RMSE, where the generalization ability was computed as the average error of all performed *K* tests:

(A6) $$G\left(M\right)=\frac{1}{K}\overset{K}{\underset{i=1}{\sum }}∥x_{i}-x_{i}^{\prime }{\left(M\right)∥}^{2}$$

The value of *K* was set to the total number of patients. The specificity was computed by creating 10,000 artificial instances (*N*) and matching these artificial instances with instances in the given dataset. The artificial instance was computed as:

(A7) $$x_{j}\left(M\right)=\bar{x}+\overset{M}{\underset{m=1}{\sum }}b_{m}\phi_{m}\;$$

This matching is performed by computing the RMSE between the artificial instance and the most similar instance in the dataset. The specificity is computed as the average error of all generated *N* shape instances:

(A8) $$S\left(M\right)=\frac{1}{N}\overset{N}{\underset{j=1}{\sum }}∥x_{j}\left(M\right)-x_{j}^{\prime }{∥}^{2}$$

with $x_{j}$ as the artificial created shape instance and $x_{j}^{\prime }$ as the most similar shape from the dataset [24].

To describe 95% and 98% of the total variation in the dataset, five and nine principal components (PCs) are needed, respectively (Figure 3 in main manuscript). The mean generalization of the SSM, including five PCs, is 3.6 (95% CI: 3.2–3.9) mm (Figure A1). The SSM, including five PCs, is able to produce an artificial neck shape with a specificity of 4.8 (95% CI: 4.7–4.8) mm (Figure A2).

## Appendix C

Distributions of the principal component analysis (PCA) scores were inspected to check what range of standard deviations from the mean were present in our dataset (Figure A3). The distribution of the PCA scores of principal component (PC) 1 showed a non-normal distribution (Figure A3a). The distribution is skewed, and values smaller than −1.5 standard deviation (SD) are not present in the histogram. The lowest value of PC 1 in our dataset was −1.3 SD. This PC denotes neck length, and scores < −1.5 SD correspond to negative neck length. Since negative neck lengths are anatomically unfeasible, it was decided to show only the shape variation from −1 SD to +3 SD. PCs 2 to 5 did not show such a skewed distribution (Figure A3b–e). Therefore, visualization of −3 SD to +3 SD shapes for these PCs were considered justified.
